# Disease and Benevolence

**Published:** 1882-07

**Authors:** 


### DISEASE AND BENEVOLENCE.

POVERTY, disease, and crime herd together, while thrift, health, and position, are found associated in the same individual. The education of the street leads to the former, the education of the church leads to the latter; by which we mean, that as a matter of civil polity, the most certain exterminator of disease, and crime, and shiftless poverty, is securing to children the benefits of a religious training, of Sunday church-going, of ministerial visitation, and of daily parental counsel on church topics.

Of the two, a thorough collegiate education, with advantages of an European tour, and the being taught to read the Bible by a pious mother and there stop, we have no hesitation in our own mind, in choosing the latter for every child of ours, if we had the option of but one of the two, and would feel an infinitely greater confidence that those children, whether boys or girls, would grow up to be independent, useful, and solid members of society.

It is a common and strong mode of making an assertion, especially in the mouths of wicked men and superficial observers, "How is it that ministers' children are worse than other people's," or, "I have always heard it said that ministers' children are the worst in the world." The sentiment seems to be a conceded fact. But it is utterly destitute of foundation. The son of a minister becoming a derelict, will attract more attention than a thousand equal criminals from the masses; hence the saying.

So far from its being true that the children of ministers, or to have a broader foundation, the children of church members, are worse than other children, it is the very reverse, and, as investigation will show, remarkably so.

A great many pointless sarcasms are thrown out in a certain class of newspapers against "Sunday religion," "Sunday Christians," "church goers," and "being devout on the Sabbath day." It certainly is better to be devout, to be externally religious, one day in a week, than not to be devout at all; better to manifest an external regard for the worship of the Almighty on a Sunday, than to throw off all obligation, and never exhibit anything of the kind.

But, after all, who are the charitable men and women of the times? With here and there a rare exception, they are church-going people, whose habitual practice is to worship in the house [illegible]

We look in vain for the erection of a hospital, the building of a college, the foundation of a professorship, and the institution of beneficiary operations from successful stage actors, play writers, gamblers, horse-racers, and circus riders. But turn to the church-going, Sabbath-abiding merchant, manufacturer, mechanic, farmer, civilian, and you will see in them the friends of good order, the patrons of the deserving, the founders of schools, the builders of churches, and the leading and patron spirits of almost every charitable institution in the land

